# Enhanced Performance by Enlarged Nano-pores of Holly Leaf-derived Lamellar Carbon for Sodium-ion Battery Anode

**DOI:** 10.1038/srep26246

**Published:** 2016-05-18

**Authors:** Peng Zheng, Ting Liu, Xiaoyan Yuan, Lifeng Zhang, Yi Liu, Jianfeng Huang, Shouwu Guo

**Affiliations:** 1 School of Materials Science and Engineering, Shaanxi University of Science and Technology, Xian 710021, Shaanxi, P. R. China; 2 Department of Electronic Engineering, School of Electronic Information and Electrical Engineering, Shanghai Jiao Tong University, Shanghai 200240, P. R. China

## Abstract

Lamellar hard carbon derived from holly leaf with enlarged pores of tiny graphite-like domains and meso-pores was prepared by hydrothermal followed high temperature pyrolysis process. Benefiting from the enlarged nano-pores of tiny graphite-like domains and the thin sheet structure with meso-pores, the derived carbon delivered a high reversible capacity of 318 mAh g^−1^ at a current rate of 20 mA g^−1^ and excellent rate capability as the anode of sodium-ion battery. And the hydrothermal followed high temperature pyrolysis method was also confirmed an effective approach for betula platyphylla and sophora japonica leaf as precursor respectively to synthesis hard carbon of lamellar structure with enlarged nano-pores of tiny graphite-like domains.

Lithium-ion batteries (LIBs) have been successfully applied into portable electronic equipment form 1990 of its first commercialization by Sony^1^. Up to now, the increasing demand as large-scale energy storage and limit natural abundance make it face inevitable challenges^2^,^3^,^4^. As one of the substitute, room-temperature rechargeable sodium-ion batteries (SIBs) have attracted tremendous attentions because of abundant Na resources around the world and its low cost^5^,^6^,^7^. SIBs have the similar work principle with LIBs as rocking-chair batteries, but the lager ionic radius of Na (55% larger than Li) demands more rigorous electrodes for capacity and stability^8^. And most of LIBs host electrodes show bad performance for SIBs, hence new electrodes with larger inserted space needed to be explored.

Carbon-based anode materials are promising candidates for SIBs due to their high conductivity, structural stability and large interlayer space available. Reduced graphene oxides^9^,^10^, carbon fibers^11^,^12^, carbon spheres^13^,^14^ and graphite^15^ have been given intensive investigation. The main strategies for afore-mentioned materials are hierarchically pores structures or enlarged interlayer lattice distance which favor for the Na-ion transport and storage. Biomass-derived hard carbon as the anode of SIBs also received substantial attention due to their easy obtain, low cost, abundant pore structure, and well performance^16^,^17^,^18^,^19^,^20^,^21^. For example, Qian *et al.*^22^ demonstrated that chicken eggshell-derived carbon fiber with hierarchical pores structure delivered a high capacity; Huang *et al.*^23^ showed pomelo peels-derived porous hard carbon offered a high capacity due to the three-dimensional (3D) porous structure and the O-functionalized surface of carbon material; Mitlin *et al.*^24^ discovered that peat moss-derived 3D macro-porous interconnected networks of carbon nanosheets presented a high capacity which was attributed to dilated graphene interlayer spacing of pseudo-graphic structures. As demonstrated by the above cases, biomass precursors have diverse structure and texture, after appropriate treatment by taking advantage of the cross-linked organization of the polymers, the derived carbon could be with well pores structure and show better performance.

As reported^25^,^26^,^27^ that natural leaves have the 3D hierarchical pores structure which is advantage for Na-ion diffusion and the integral connected carbon framework is beneficial for electro transfer. The biomass-derived hard carbons have large interlayer space which is suitable for Na-ion insertion. Holly (*Buxus megistophylla Levl.*) has worldwide distribution in temperature zones, which mainly cultivated in the Middle and South America and tropical Asiatic. It could grow all of the year without any special maintaining. So it has tremendous production. Motived by its special porous structure and reuse of low cost precursor, there is much attracting to probe whether holly leaf-derived carbons is suitable as the anode of SIBs. We found the holly leaf-derived two-dimensional (2D) hard carbon with enlarged pores of tiny graphite-like domains was synthesized by hydrothermal carbonization followed pyrolysis process, which exhibited high discharge capacity of 253 mA h g^−1^ at 20 mA g^−1^ after 100 cycles, well rate and cycling performance. However, if only deal with the high temperature pyrolysis, the product was bulk with the common pores of tiny graphite-like domains^28^,^29^,^30^ and showed bad performance for SIBs. In order to further confirm the enlarged nano-pores could be produced by hydrothermal carbonization followed pyrolysis process, nature leaf such as, betula platyphylla and sophora japonica leaf were additional treated as the start precursor respectively, and the same result was obtained as that of holly leaf.

## Experimental

### Synthesis of Hard Carbon Samples

#### Materials

The precursor of holly, betula platyphylla and sophora japonica grew in the campus of Shaanxi University of Science and Technology (Shaanxi, P. R. China). Before carbonizations, the precursor leaves were thoroughly cleaned using deionized water and EtOH, dried at room temperature and cut into small piece.

#### Fabrication of HTPC-H (B/S)

For carbonizing the precursor leaves through hydrothermal process followed high temperature pyrolysis, typically, 3 g precursor leaf and 35 mL deionized water were placed in a 50 mL Teflon-lined autoclave and heated at 140, 160, 180 and 200 °C for 10 h firstly, respectively. After being cooled to room temperature naturally, the solid product was collected, washed with deionized water and 3 M HCl, and then dried at 353 K for 12 h, which referred to as HTC-H(B/S)-x (H, B and S stands for the precursor of holly leaf, betula platyphylla leaf and sophora japonica leaf, respectively and x represents the carbonization temperature). Then, the HTC-H-x was annealed further at 800 °C (heating rate, 5 °C min^−1^) for 2 h under Ar atmosphere in a tubular furnace, and the final product was labeled as HTPC-H(B/S)-x.

#### Preparation of DPC-H (B/S)

For direct pyrolysis carbonization, typically, 5 g of precursor leaf was loaded in a tubular furnace and heated at 800 °C for 2 h under Ar atmosphere. The solid product was washed by 3 M HCl to remove the impurity of calcium, then washed by deionized water till the pH of 7 about. The sample was finally dried overnight at 60 °C for 12 h in an oven. The as-obtained product was denoted as DPC-H (B/S) for the precursor of holly leaf, betula platyphylla leaf and sophora japonica leaf, respectively.

### Materials Characterization

The structural characteristics of the samples were investigated using X-ray powder diffraction (XRD) (Rigaku D/MAX2200PC) at room temperature with Cu Kα (λ = 1.54178 Å) radiation and Raman spectrometer (Raman- Invia) with a 532 nm excitation laser. The elemental compositions of the samples were investigated using X-ray photoelectron spectrometer (XPS) (ULVAC-PHI5000). The morphology of the samples were confirmed by scanning electron microscopy (SEM) (Rigaku S4800) and transmission electron microscopy (TEM) (FEI Tecnai F20 microscope). For observing the morphology of pristine holly leaf precursor and HTC, the leaf was dried at 80 °C for 10 h under vacuum, and then grinded into powder. The specific surface area was calculated with the Brunauer–Emmett–Teller (BET) model, and the pore-size distribution was calculated from the adsorption/desorption data by using the Density Functional Theory (DFT) method.

### Electrode fabrication and electrochemical measurements

The two-electrode electrochemical test 2032 coin cells were assembled in an argon-filled glove box using the as-prepared carbon materials as anodes (the working electrode) and sodium pellets as the counter electrode. The working electrode was prepared by mixing the as-obtained carbon materials, conducting agent (Super P), and polyvinylidene fluoride (PVDF) (in N-methyl-2-pyrrolidone) with a weight ratio of 80:10:10. The obtained slurry was coated onto a copper foil and dried at 110 °C for 10 h under vacuum. The electrolyte was 1 M NaClO_4_ in 1:1 by volume ethylene carbonate (EC) and diethyl carbonate (DEC). Celgard 2500 membrane/glass fibers was the separator. The charge–discharge tests were performed on a Newaresles battery test system (BTS) (Shenzhen, China) in a voltage range of 0.01–3.0 V at different current rates. The capacity was calculated based on the mass of active material. Cyclic voltammetry (CV) testing at a scan rate of 0.1 mV s^−1^ between 0.01 and 3.0 V was performed on a CHI660E electrochemical workstation (Chenhua Instrument Company, Shanghai, China). Electrochemical impedance spectra (EIS) measurements were carried out in the frequency range from 100 kHz to 0.01 Hz on a CHI 660E electrochemical workstation.

## Results and Discussion

**Figure 1:**
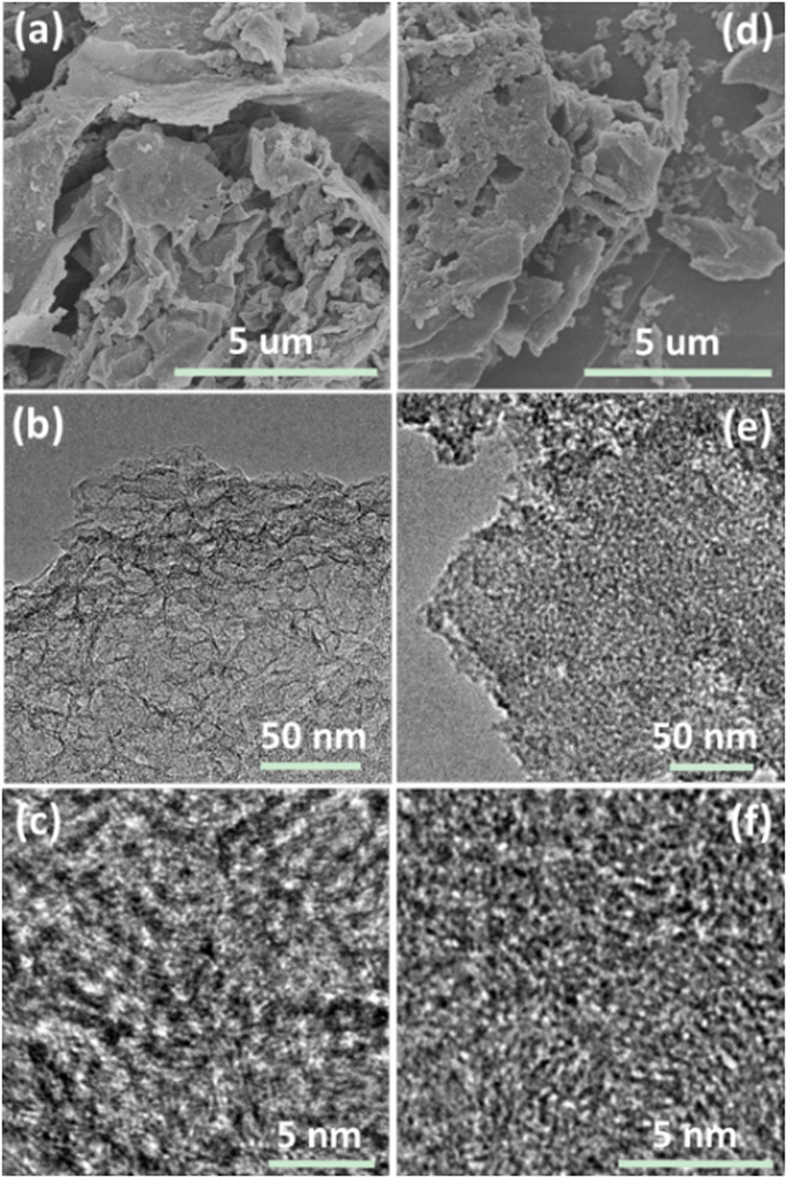
SEM (**a**), TEM (**b**) and HRTEM (**c**) images of HTPC-H-160; and SEM (**d**), TEM (**e**) and HRTEM (**f**) images of DPC-H.

Holly leaf-derived hard carbon of lamellar structure with enlarged nano-pores of tiny graphite-like domains and meso-pores was prepared by hydrothermal carbonization followed pyrolysis process. Figure S1a shows the scanning electron microscopy (SEM) image of pristine holly leaf precursor, it has lots of hierarchical pores as we had been expected. The holly leaf precursor was first conducted hydrothermal treatment under 160 °C for 10 h and the product is named as HTC-H-160. The color of holly leaf changed into brown form green (inset of Fig. S1a,b). As shown in Fig. S1b,c, HTC-H-160 has sheet morphology. The corresponding high resolution transmission electron microscopy (HRTEM) image of HTC-H-160 is shown by Fig. S1d, it is amorphous and has common nano-pores of tiny graphite-like domains^28^,^29^,^30^. Subsequently, HTC-H-160 sample was pyrolyzed at 800 °C for 2 h under Ar atmosphere, the as-obtained product is called HTPC-H-160 and the brown leaf turned into black powder. The microstructure of HTPC-H-160 is shown in Fig. 1a–c. HTPC-H-160 is still kept as lamellar structure (Fig. 1a) and the thickness is ~20 nm. From the transmission electron microscopy (TEM) (Fig. 1b), many of meso-pores are observed, which could also be confirmed by the BET result (Fig. S2). It is still amorphous but the nano-pores of tiny graphite-like domains are enlarged (Fig. 1c). The size of the nano-pore is 1.1 nm about. However, when the holly precursor was direct pyrolyzed at 800 °C for 2 h under Ar atmosphere without hydrothermal treatment (denoted as DPC-H), the morphology and microstructure is obviously different with that of HTPC-H-160. The main parts of DPC-H are bulks with the size of ~3 um and some small particles appeared (Fig. 1d). The bulk has compacted structure (Fig. 1e) and little meso-pore is observed. Figure 1f presents the HRTEM image of DPC-H, it is also amorphous but the size of the nano-pores is ~0.5 nm which is much smaller than that of HTPC-H-160. The bulk morphology and the nano-pores of graphite-like domains of DPC-H are consistent with the common results^28^,^29^,^30^.

**Figure 2:**
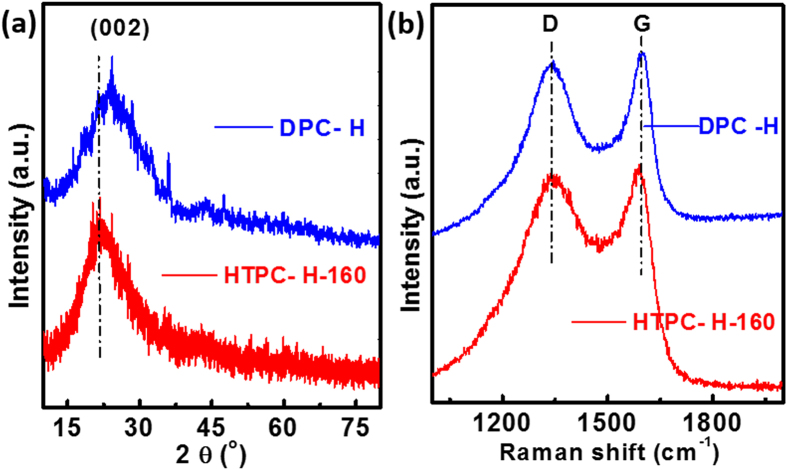
(**a**) XRD patterns and (**b**) Raman spectra for HTPC-H-160 and DPC-H.

The amorphous structure of HTPC-H-160 and DPC-H is further verified by X-ray diffraction (XRD) technique. Figure 2a shows the XRD patterns of the HTPC-H-160 and DPC-H. The holly leaf precursor contains some calcium compounds impurities, which were removed by 3 M HCl. Both of HTPC-H-160 and DPC-H have broad diffraction peaks at around 24° ((002) crystallographic planes of graphite). And peaks at 24° of HTPC-H-160 shift toward low degree compared with the XRD spectra of DPC-H, which indicates that HTPC-H-160 has larger layer distance than that of DPC-H, but both of them are still amorphous carbon. The degree of graphitization of HTPC-H-160 and DPC-H was also investigated by Raman spectrum. As shown in Fig. 2b, two characteristic peaks around 1340 and 1590 cm^−1^ are indexed to the D (disordered carbon or defective graphitic structures) and G (vibration of sp2-bonded carbon atoms in a 2D hexagonal lattice and) bands, the I_G_/I_D_ ratios for HTPC-H and DPC-H are 1.05 and 1.04, respectively. This is consistent with the XRD result, due to cellulose and hemicellulose of holly leaf precursor are not easy to graphitization. Figure S3 shows the X-ray photoelectron spectroscopy (XPS) spectra of HTPC-H-160 and DPC-H. They have the same compositions and the primary constitutions are C and O, and the N1s signal is rather weak for HTPC-H-160 and DPC-H.

**Figure 3:**
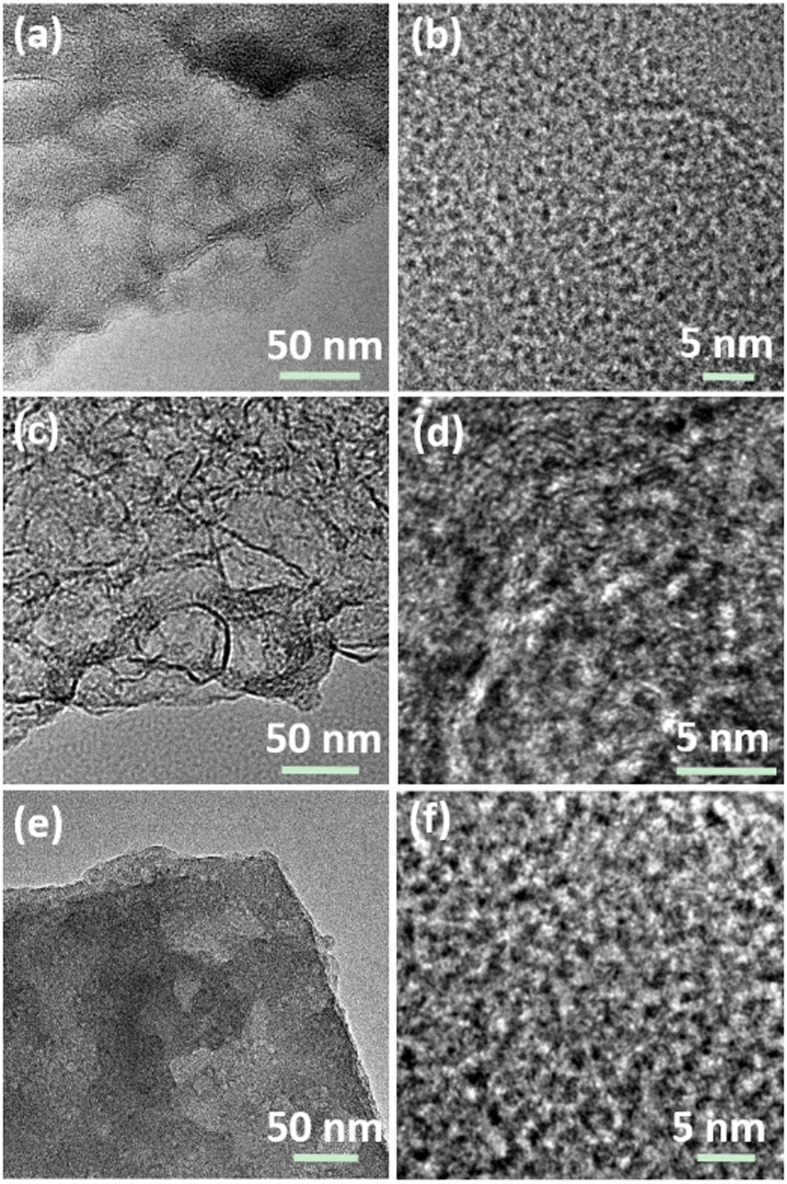
(**a** and **b**), (**c** and **d**) and (**e** and **f**) HRTEM images for HTPC-H-140, HTPC-H-180 and HTPC-H-200, respectively.

To investigate the effect of temperature on the thickness of sheet and the size of nano-pores for HTPC-H, at first, the holly leaf were hydrothermal treated at 140, 180 and 200 °C, respectively, and followed the same pyrolysis process at 800 °C, the structure of the products are shown Fig. 3. At the 140 °C hydrothermal treatment followed pyrolysis, the obtained derived carbon is thin sheet, but it has the common nano-pores of tiny graphite-like domains (Fig. 3a,b). However, the porous sheet morphology with enlarged nano-proes is gained at 180 °C after pyrolysis, as indicated by the HTREM images of Fig. 3c,d. With the hydrothermal temperature increased to 200 °C, the pyrolysis sheet become compact, but the nano-pores of tiny graphite-like domains are further enlarged (Fig. 3e,f). Thus, the hydrothermal treatment temperature has primary effect on the final structure. Then the HTC-180 sample was further annealed at 1000 and 1200 °C, respectively, the structure has no change.

Although the phase structure and the chemistry composition of HTPC-H and DPC-H have no difference, the morphology for HTPC-H and DPC-H were totally different, one is lamellar with meso-pores and the other is bulk, and HTPC-H have larger nano-pores of tiny graphite-like domains than that of DPC-H. The holly leaf precursor was composed of cellulose, hemicellulose and lignin^31^,^32^, the tissue is highly heterogeneous and they are less liable to graphite even under harsh conditions. During the mild hydrothermal carbonization process, light-level of cross-linking (dehydration, condensation and carbonization) are happened^33^. So, the hierarchical pores of holly leaf precursor are vanished, but the framework of lamellar structure is kept which was mainly composed of cellulose-carbonization. We argued that during the formation of HTC-H procedure, besides the cross-linking for light substances and heavy substances respectively, there is also the light substances cross-linking with the heavy substances, which could be reflected by the rough and smooth surface of HTC-H (Fig. S4). During the followed pyrolysis at 800 °C processes, heavy-level of cross-linking occurred, and lots of light substances are decomposed, so the meso-pores are emerged and the nano-pores of tiny graphite-like domains are enlarged. The separated different degrees of carbonization of hydrothermal and further high temperature pyrolysis play a key role for these, relaxed cross-linking happened during the two processes and much more substances are decomposed in the enough time and space compared with that of DPC-H, the yield of HTPC-H-160 is 16.7% and that for DPC-H is 22.8%, which could be proved by the thermogravimetric analysis (TGA) (Fig. S5). The resulted enlarged nano-pores and meso-pores are favor for the diffusion and storage of large radius of Na-ion.

**Figure 4:**
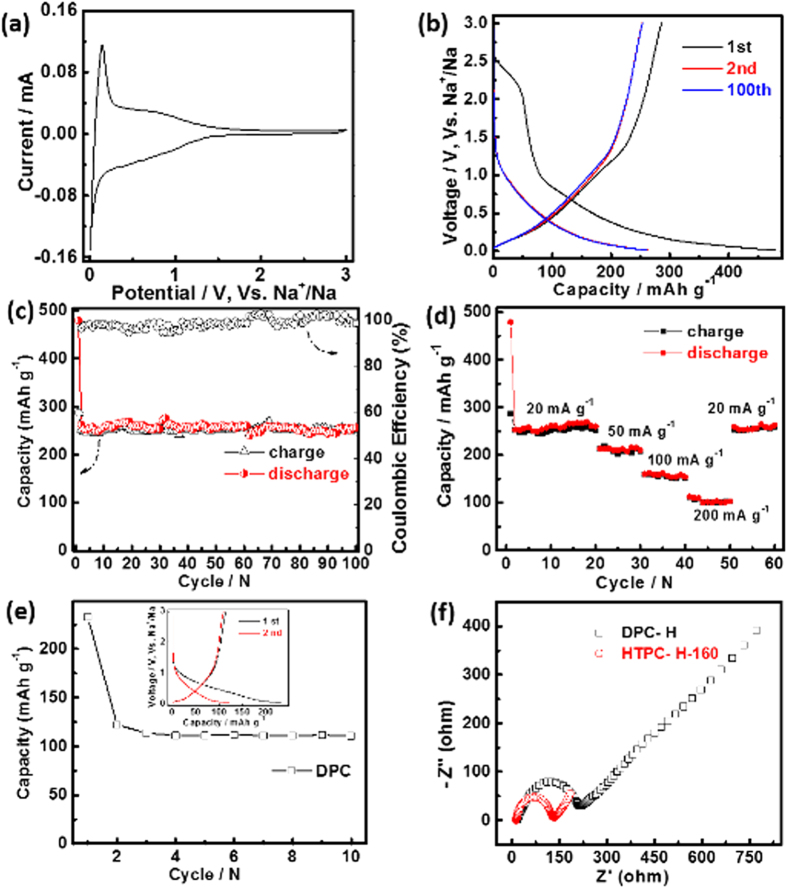
(**a**) CV curve of HTPC-H-160 tested at 0.1 mV s^−1^, (**b**) Discharge–charge curves of HTPC-H-160 at 20 mA g^−1^; (**c**) Cycling performance of HTPC-H-160 at the current densities of 20 mA g^−1^; (**d**) Rate performance of HTPC-H-160 at the current densities of 20, 50, 100, 200 and 20 mA g^−1^. (**e**) Cycling performance of DPC-H at the current densities of 20 mA g^−1^, the inset is the discharge–charge curves; (**f**) Nyquist plots of HTPC-H-160 and DPC-H electrode.

To probe the electrochemical performance of the derived carbons as the anode for SIBs, cyclic voltammetry (CV) and galvanostatic discharge/charge cycling were performed in a sodium half-cell. Figure 4a presents CV curve for HTPC-H-160 anode at a scanning rate of 0.1 mV s^−1^ in the range of 0.1–3 V. A pair of redox peaks raised over a wide voltage region (0.1–0.5 V) in both the cathodic and the anodic portions. The humps are ascribed to the insertion/extraction of Na-ion in the hard carbon which corresponds to the sloping regions of the galvanostatic discharge/charge profiles (Fig. 4b)^24^,^34^. The HTPC-H-160 anode delivers a high reversible capacity of 479, 262 and 253 mAh g^−1^ for 1st, 2nd and 100th cycle at 20 mA g^−1^, respectively. During all of the cycles, the HTPC-H-160 anode shows the primary slop region except the 1st cycle in which the low-potential plateau region is accounted for the formation of solid electrolyte interface (SEI) film. And the slop potential profile is attributed to insertion of sodium between parallel or nearly parallel layers^21^,^35^. According to the literature report, most of the capacity for hard carbon derived from the low-potential region (around 0 V) close to the Na plating potential, which may cause safety issues during fast charging process^23^. However, in this report, the main capacity derived from slop region and the detailed capacity ratios of slop region and low-potential region could be seen Fig. S6. Figure 4c shows the detailed cycling performance evolution at 20 mA g^−1^, at the end of 100th cycle the HTPC-H-160 anode arrives a reversible capacity of 253 mAh g^−1^ which kept 99% of its 2nd discharge capacity (254 mAh g^−1^). And the first-cycle Coulombic efficiency is 60%, in the subsequent cycles, the Coulombic efficiency rises dramatically to 97%. Figure 4d exhibits rate capabilities of the HTPC-H-160 electrodes form 20 mA g^−1^ to 200 mA g^−1^. With increasing charge-discharge rate, the discharge capacity of HTPC-H-160 electrode is reduced to 210, 155 and 103 mAh g^−1^ at the rates of 50, 100 and 200 mA g^−1^, respectively. As the current density is decreased to 20 mA g^−1^, the capacity recovered to 252 mAh g^−1^ again. The well cycling and rate performance benefit from the enlarged nano-pores of tiny graphite-like domains and thin lamellar structure with meso-pores. However, the bulk of DPC-H with common nano-pores of tiny graphite-like domains shows poor discharge capacity of 112 mAh g^−1^ at 20 mA g^−1^ (Fig. 4e), which is much smaller than that of HTPC-H-160. The enlarged nano-pores of tiny graphite-like domains of HTPC-H-160 compared to that of DPC-H play an important role for Na-ion diffusion. Electrochemical impedance spectroscopy (EIS) was employed to study the electrochemical kinetics of the derived carbons. Figure 4f shows the Nyquist plots of the carbons electrodes. Compared with DPC-H anode, HTPC-H-160 anode has smaller charge transfer resistance, which guarantees good rate performance and further confirms the advantage of enlarged nano-pores.

**Figure 5:**
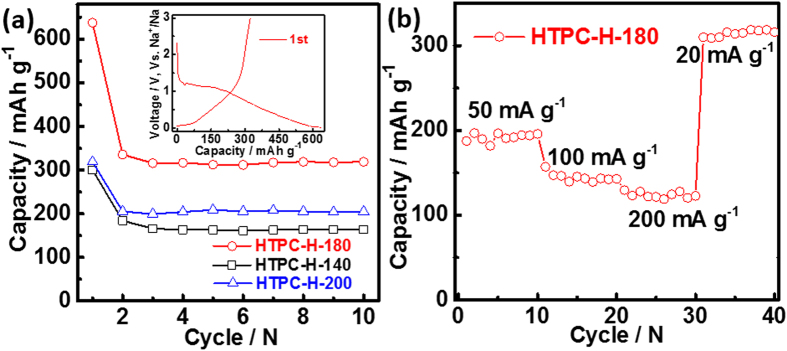
(**a**) Cycling performance of HTPC-H-140, HTPC-H-180 and HTPC-H-200 electrode at the current densities of 20 mA g^−1^, the inset is discharge–charge curves of HTPC-H-180 at 20 mA g^−1^; (**b**) Rate performance of HTPC-H-180 at the current densities of 50, 100, 200 and 20 mA g^−1^.

The electrochemical performance of the samples with different hydrothermal treatment temperatures followed pyrolysis at 800 °C were also investigated as the anode for SIBs in the coin cell. As shown by Fig. 5a, at the end of 10th cycle, the discharge capacities are 164, 318 and 204 mAh g^−1^ at 20 mA g^−1^ for HTPC-H-140, HTPC-H-180 and HTPC-H-200, respectively. HTPC-H-180 shows the higher capacity than that of HTPC-H-160, the reason is ascribed to that HTPC-H-180 has more micropore than that of HTPC-H-160 (Fig. S2), which are beneficial for Na-ion storage. In comparison, the common nano-pores and compact sheet have negative effect on Na-ion transport, so their capacities are reduced. The first-cycle Coulombic efficiency of HTPC-H-180 is 50%, which is smaller than that of HTPC-H-160 (inset of Fig. 5a). Figure 5b exhibits the excellent rate performance of HTPC-H-180, at the current density of 50, 100, 200 mA g^−1^, the discharge capacities could be arrived at 190, 140 and 127 mAh g^−1^, respectively. When the current density is shifted to 20 mA g^−1^, the capacity is restored to 318 mAh g^−1^ again. Such outstanding rate performance is also resulted from its unique structure.

In order to further confirm that whether the hydrothermal treatment followed pyrolysis carbonization process is a general method to enlarge the nano-pores of tiny graphite-like domains for nature leaf, betula platyphylla and sophora japonica leaf were chose as precursors respectively. The results are shown by Figs S7 and S8. HTPC-B-160 (Fig. S7a,b) and HTPC-S-160 (Fig. S8a,b) also have lamellar structure with enlarged nano-pores of tiny graphite-like domains (Figs S7c and S8c), while DPC-B (Fig. S7d) and DPC-S (Fig. S8d) are the bulk with the common nano-pores of tiny graphite-like domains (Figs S7e and S8e). As the anode of NIBs, the capacities of HTPC (−B/S)-160 are higher than that of DPC (−B/S) (Figs S7f and S8f), but lower than the capacity of HTPC-H-160. It is due to the absence of meso-porous and more compact stack of HTPC-B (/S)-160 sheets than that of HTPC-H-160, which are not favor for the transfer of Na-ion in the interlayer of hard carbons.

## Conclusions

In summary, we had prepared holly leaf-derived hard carbon (HTPC-H-160/180) with lamellar structure comprised of enlarged nano-pores of tiny graphite-like domains and meso-pores by hydrothermal treatment followed by pyrolysis process. The nano-pores of tiny graphite-like domains of HTPC-H-160/180 is ~2 times larger than that of DPC-H which was synthetized by direct pyrolysis treatment. Such large nano-pores are advantage for Na-ion transfer and storage. HTPC-H-180 delivers a high reversible capacity of 318 mAh g^−1^ at a current rate of 20 mA g^−1^ and excellent rate capability. And it had been demonstrated that the hydrothermal treatment followed high temperature pyrolysis method was an effective approach for preparation of hard carbon materials with lamellar structure and enlarged nano-pores using betula platyphylla and sophora japonica leaf as raw materials respectively. Considering the superior electrochemistry performance and the low cost, holly leaf-derived HTPC-H-180 hard carbon would be a competitive anode for SIBs.

## Additional Information

**How to cite this article**: Zheng, P. *et al.* Enhanced Performance by Enlarged Nano-pores of Holly Leaf-derived Lamellar Carbon for Sodium-ion Battery Anode. *Sci. Rep.* **6**, 26246; doi: 10.1038/srep26246 (2016).

## Supplementary Material

Supplementary Information
