# Supplementary material for: Enhanced Performance by Enlarged Nano-pores of Holly Leaf-derived Lamellar Carbon for Sodium-ion Battery Anode
Source: Sci Rep. 2016 May 18;6:26246. doi: 10.1038/srep26246 (PMC4870559; doi:10.1038/srep26246)
Supplement: Supplementary Information [file srep26246-s1.doc]

**Supplementary data**

**Enhanced Performance by Enlarged Nano-pores of Holly Leaf-derived Lamellar Carbon for** **Sodium-ion Battery Anode**

Peng Zheng,^*a^ Ting Liu,^a^ Xiaoyan Yuan,^a^ Lifeng Zhang,^a^ Yi Liu,^a^ Jianfeng Huang, ^*a^ and Shouwu Guo, ^*a, b^

*^a^* School of Materials Science and Engineering, Shaanxi University of Science and Technology, Xian 710021, Shaanxi, P. R. China.

*^b^* Department of Electronic Engineering, School of Electronic Information and Electrical Engineering, Shanghai Jiao Tong University, Shanghai 200240, P. R. China

ZhengPeng@sust.edu.cn； huangjf@sust.edu.cn； swguo@sjtu.edu.cn


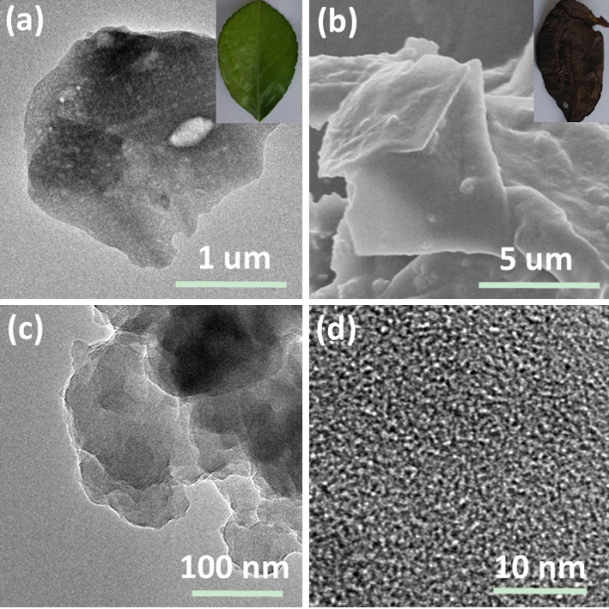


**Figure S1.** (a) TEM image of holly leaf precursor, the inset is macroscopic photograph of holly leaf precursor; (b) SEM, (c) TEM and (d) HRTEM images of HTC-H-160, inset of (b) is macroscopic photograph of HTC-H-160.


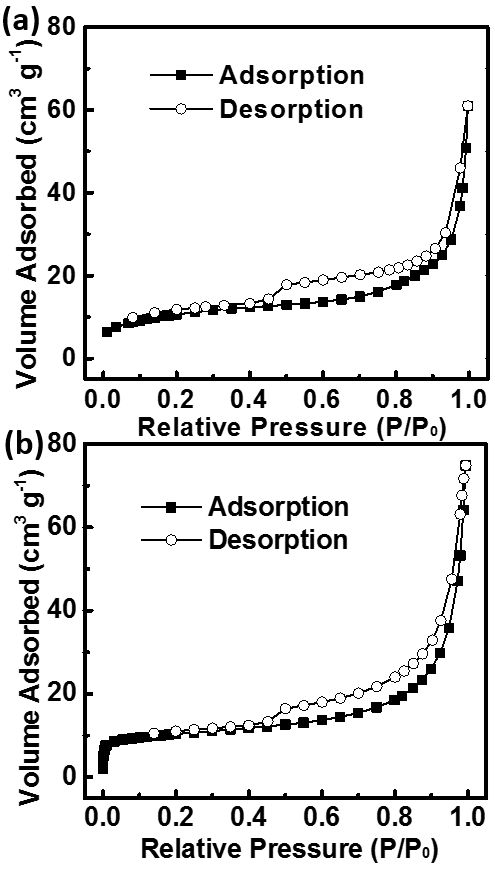


**Figure S2.** (a, b) Nitrogen adsorption/desorption isotherms for the HTPC-H-160 and HTPC-H-180, respectively.


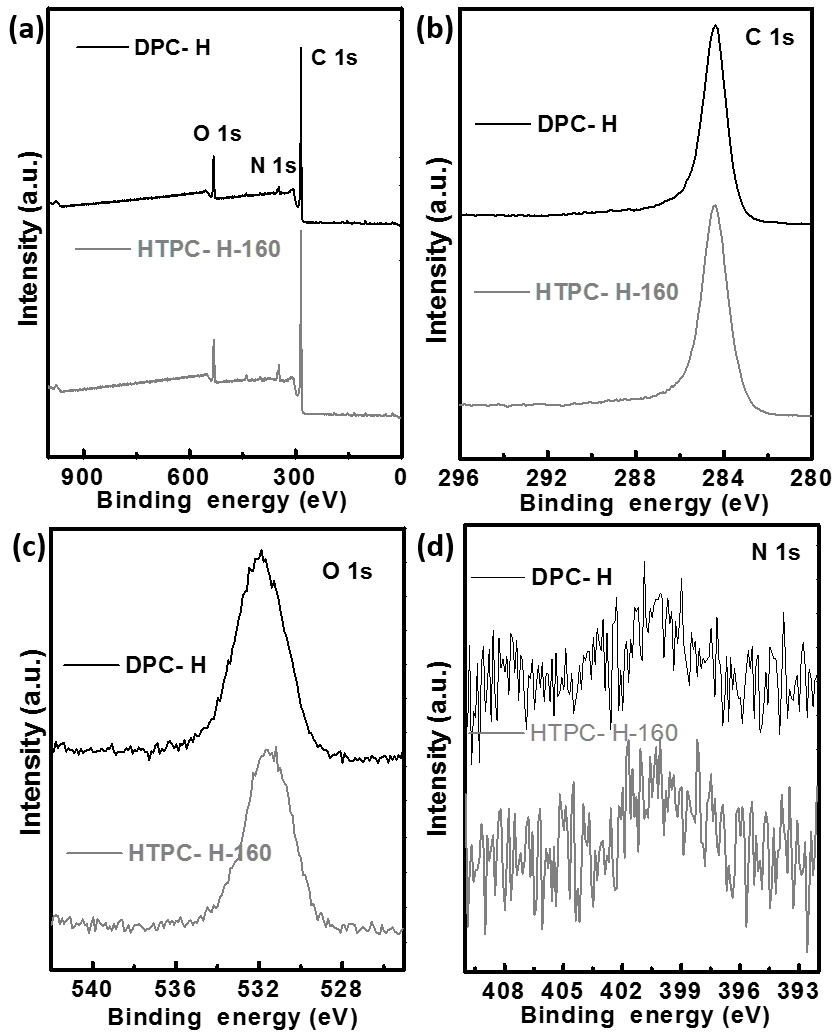


**Figure S3.** XPS spectra of HTPC-H-160 and DPC-H composites for full survey (a), N 1s(b), O 1s (c) and C 1s (d) spectra.


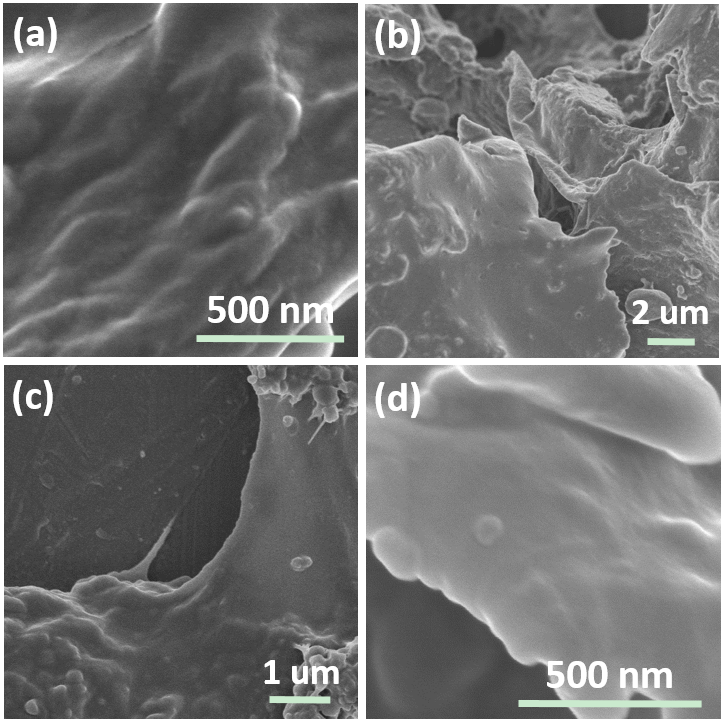


**Figure S4.** (a- d) FESEM images for HTC-H-140, HTC-H-160, HTC-H-180 and HTC-H-200, respectively.


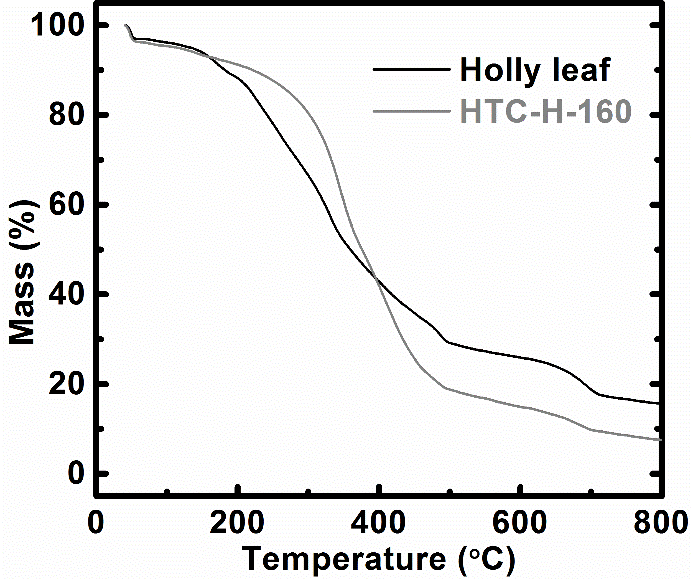


**Figure S5.** Thermogravimetric analysis of holly leaf and HTC-H-160.


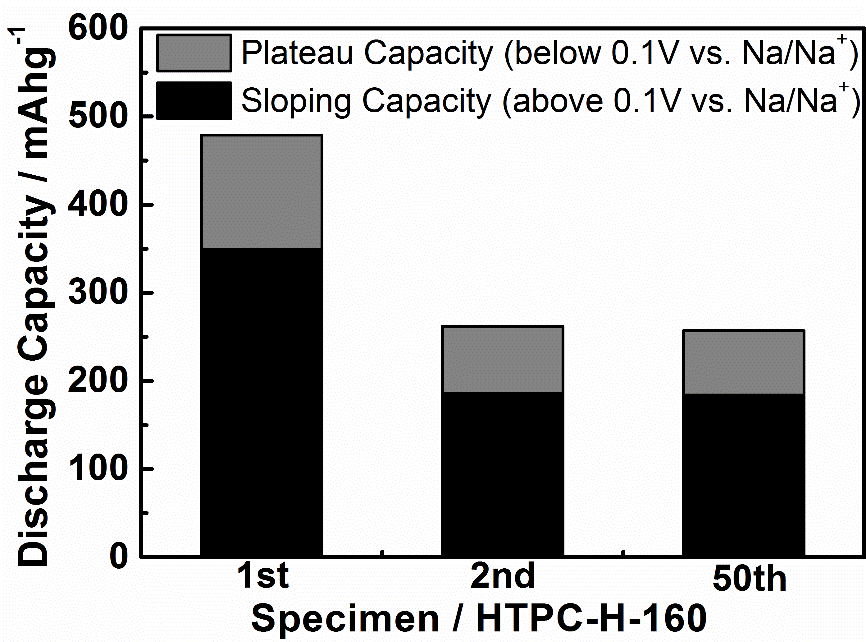


**Figure S6.** Summary of capacity potential distribution of HTPC-H-160 electrodes for 1st, 2nd and 10th cycle at current density of 20 mA g^-1^.


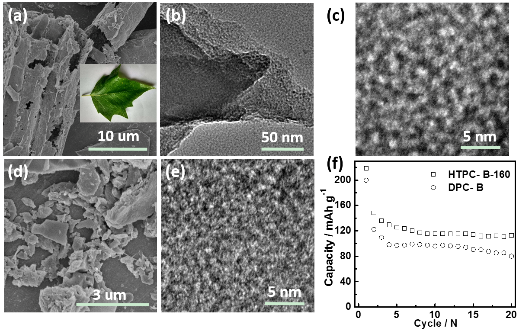


**Figure S7.** Betula platyphylla leaf-derived carbon: (a) SEM image of HTPC-B-160, the inset is macroscopic photograph of Betula platyphylla; (b) TEM, and (c) HRTEM images of HPTC-B-160, (d) SEM image of DPC-B, (e) HRTEM images of DPC-B, (f) Cycling performance of HTPC-B-160 and DPC-B at the current densities of 20 mA g^-1^.


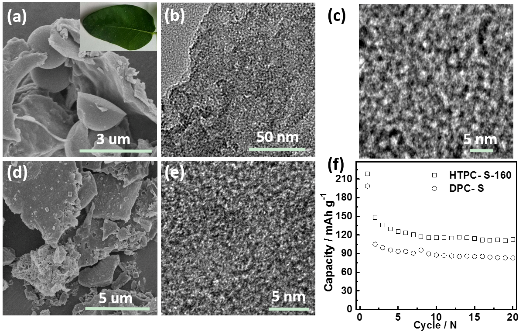


**Figure S8.** Sophora japonica leaf-derived carbon: (a) SEM image of HTPC-S-160, the inset is macroscopic photograph of Sophora japonica-S; (b) TEM, and (c) HRTEM images of HPTC-S-160, (d) SEM image of DPC-S, (e) HRTEM images of DPC-S, (f) Cycling performance of HTPC-S-160 and DPC-S at the current densities of 20 mA g^-1^.
